# What Impacts Perceived Stress among Canadian Farmers? A Mixed-Methods Analysis

**DOI:** 10.3390/ijerph18147366

**Published:** 2021-07-09

**Authors:** Briana N. M. Hagen, Alex Sawatzky, Sherilee L. Harper, Terri L. O’Sullivan, Andria Jones-Bitton

**Affiliations:** 1 Department of Population Medicine, University of Guelph, 50 Stone Road East, Guelph, ON N1G 2W1, Canada; as@alex-sawatzky.com (A.S.); tosulliv@uoguelph.ca (T.L.O.); aqjones@uoguelph.ca (A.J.-B.); 2 School of Public Health, University of Alberta, 3-300 Edmonton Clinic Health Academy, 11405-87 Avenue, Edmonton, AB T7G 1C9, Canada; sherilee.harper@ualberta.ca

**Keywords:** stress, farmers, mental health, agriculture, mixed methods

## Abstract

Globally, farmers report high levels of occupational stress. The purpose of this study was to identify and explore factors associated with perceived stress among Canadian farmers. A sequential explanatory mixed-methods design was used. An online cross-sectional national survey of Canadian farmers (n = 1132) was conducted in 2015–2016 to collect data on mental health, demographic, lifestyle, and farming characteristics; stress was measured using the Perceived Stress Scale. A multivariable linear regression model was used to investigate the factors associated with perceived stress score. Qualitative interviews (n = 75) were conducted in 2017–2018 with farmers and agricultural sector workers in Ontario, Canada, to explore the lived experience of stress. The qualitative interview data were analyzed via thematic analysis and then used to explain and provide depth to the quantitative results. Financial stress (highest category—a lot: (*B* = 2.30; CI: 1.59, 3.00)), woman gender (*B* = 0.55; CI: 0.12, 0.99), pig farming (*B* = 1.07; CI: 0.45, 1.69), and perceived lack of support from family (*B* = 1.18; CI: 0.39, 1.98) and industry (*B* = 1.15; CI: 0.16–2.14) were positively associated with higher perceived stress scores, as were depression and anxiety (as part of an interaction). Resilience had a small negative association with perceived stress (*B* = −0.04; CI: −0.06, −0.03). Results from the qualitative analysis showed that the uncertainty around financial stress increased perceived stress. Women farmers described the unique demands and challenges they face that contributed to their overall stress. Results from this study can inform the development of mental health resources and research aimed at decreasing stress among Canadian farmers.

## 1. Introduction

There is a wide body of evidence supporting reports that high levels of psychological stress have detrimental impact on mental health, including some evidence among farming populations [1,2]. Chronic stress is reportedly positively associated with depression, anxiety, and suicide [1,3]. Results from the 2005 Canadian National Population Health Survey reported that individuals experiencing high work stress (75th percentile or higher) were 2.35 times more likely (95% CI = 1.54–3.77), compared to those below the 75th percentile, to experience a depressive episode [4].

According to the Fourth European Working Conditions Survey, 32% of agricultural and fishery workers reported that stress negatively impacted their mental and physical health, which was significantly higher than other occupations (22%) [5]. Stress among farmers has also been correlated with increased farm accidents and injury in Australia [6]. Within farming populations in Australia, high stress has been linked with elevated suicide rates [3]. Other studies with farmers in the United States and Australia have reported associations between high stress and withdrawal from community activities or social commitments, decreased self-esteem, physical health problems, increased substance use, and relationship break-downs [1,7].

A recent Canadian study reported that perceived stress was significantly higher among farmers than reported normative data [8]. While research examining what factors impact stress has been conducted among farmers in the United States [9], the United Kingdom [1], Finland [10], and Australia [11], there have been few studies investigating factors associated with perceived stress among Canadian farmers; two of the most recent published Canadian studies were conducted in 1999 [12]. Factors identified to be associated with higher stress in those 1999 studies included market variability, financial pressure, stoicism, weather, and workload [12,13]. Given the association of stress with poor mental and physical health [5], as well as the importance of Canadian agriculture to trade, employment, and economy [14], a current investigation of stress in Canada’s farming population is warranted.

Perceived stress is complex, with a multitude of contributing factors that may not be thoroughly explained through either quantitative or qualitative work alone. Use of mixed-methods approaches to explore perceived stress among farmers allows for a more comprehensive understanding of factors that influence stress than a quantitative or qualitative method alone. Mixed qualitative and quantitative method designs are being used more frequently to address health research questions [15]. Here, we use mixed quantitative and qualitative methods to explore perceived stress among a sample of Canadian farmers. The goal of this study was to identify and explore the factors associated with perceived stress among Canadian farmers.

## 2. Materials and Methods

### 2.1. Study Design

A sequential explanatory mixed-methods design [16] was used to achieve the study goal. First, quantitative methods were employed to investigate factors associated with perceived stress among farmers. Second, qualitative methods were used to further explain and deepen understanding of the quantitative model results.

### 2.2. Quantitative Methods

#### 2.2.1. Survey Recruitment

A national, cross-sectional survey investigating several mental health outcomes among farmers in Canada was conducted between September 2015 and February 2016 [8]. Sample size calculations for prevalence dictated a minimum required sample size of 385 farmers [8]. Perceived stress was measured with the Perceived Stress Scale (PSS) [8]. Demographic, lifestyle, and farming characteristics data were also collected.

The questionnaire was delivered online. National and provincial agricultural organizations were asked to share the survey link to their membership via listservs, emails, and newsletters. The survey was also promoted by these organizations via social media. All self-identified Canadian farmers over the age of 18 years who were able to read and write in English were eligible to participate in the study. Questionnaire data were collected anonymously after obtaining written informed consent. If chosen by the participant, an email address could be provided (separate from questionnaire responses) for entry into a draw for one of three cash incentives ($250).

#### 2.2.2. Survey Participants

A total of 1132 participants completed the online survey. A detailed description of survey participant demographic information has been previously reported [8]. Briefly, the study population was 69.4% men, 34.4% women, and 0.2% gender non-binary, with an average age of 46.6 years (range: 19–88). The majority of participants were married (77.8%) or in a committed relationship (9.9%); 9% of participants were single, 2.2% divorced, 0.7% widowed, and 0.5% described their relationship status as “other”. Farmers from ten provinces were represented in the sample, with participants from Ontario making up 72.5% of the total study population [8]. As previously reported, the average PSS score was 18.9 (out of a possible 24) (standard deviation (SD) = 4.9), which was notably higher than a representative normative sample of 2000 individuals from the United States (women: mean (M) = 13.7; men: M = 12.1) [8].

#### 2.2.3. Data Collection

##### The Perceived Stress Scale (PSS)

The PSS measured “how unpredictable, uncontrollable, and overloading respondents find their lives” [17]. This 10-item scale is a validated tool that is internally consistent, valid, and reliable [18] and is the most widely used measurement tool to examine stress [18]. Each PSS item was scored on a 5-point Likert scale (0–4) [18]. Scores ranged from 0–40, with higher scores indicating higher perceived stress [18]. The PSS is intended to measure stress at a specific moment in time, but it is not a diagnostic tool; the predictive ability of the PSS to accurately measure an individuals’ perceived stress may decline after 4–8 weeks [18]. Participants were asked to report based on their experiences in the previous month.

If participants had one missing value within the PSS, a mean substitution was used [8]. As consistent with previous literature [19], if a participant had two or more missing values, they were omitted from the analyses.

##### Other Data Items

Resilience scores were measured using the Connor-Davidson Resilience Scale [20]. This 24-item scale has been validated and used widely to measure resilience among the United States general population [20]. Anxiety and depression were measured using the Hospital Anxiety and Depression Scale [21]. This self-reported screening tool has been deemed valid and reliable, using distinct subscales for depression (“state of loss of interest and diminished pleasure response”) and anxiety (“restless, anxious moods and thoughts”) over the past week [20].

Demographic and farm variables to be included in the model were selected based on previous research [1,22,23] and consultation with our stakeholder working group. Included variables were: farming commodity, farm ownership (yes/no), how rural a farm was (kilometer distance from urban center), marital status (single, married or in a committed relationship, separated, divorced, widowed), and financial stress (none, a little, some, a lot). Lifestyle variables included self-rated health and mental health (poor, good, very good, excellent), past mental illness (yes/no), and perceived supports. A participant’s perceived satisfaction with support from their spouse/romantic partner, family, friends, and industry was self-reported using a Likert scale format. Participants could select that they were “not at all satisfied”, “dissatisfied”, “somewhat satisfied”, “satisfied”, or “very satisfied”. These data items were dichotomized into two groups: “satisfied” (which included those who selected “somewhat satisfied”, “satisfied”, or “very satisfied”) and “dissatisfied” (which included those who selected “not at all satisfied” or “dissatisfied”).

#### 2.2.4. Survey Analysis

The outcome variable of interest was the perceived stress score, measured using the PSS. To examine the factors associated with perceived stress, a multivariable linear regression model was built using STATA 15^©^ (Stata Corp LLC, College Station, TX, USA). Guided by previous research and a causal diagram, factors of interest were identified a priori, and data were drawn from the national survey data. Independent variables of interest were examined for collinearity using Spearman’s rank correlation coefficient [24]. A correlation of >0.80 was considered highly collinear. If two variables were highly collinear, the variable determined to be most consistent with the current research knowledge [25] and with the most complete data was used in the analysis, while the other variable was removed [24]. Continuous independent variables of interest were examined graphically using a Locally Weighted Scatterplot Smoothing (Lowess) curve for linearity with the dependent variable [24]. A hypothesis testing approach was used to build the multivariable model. First, a series of unconditional univariable linear regression analyses were conducted using a liberal *p*-value (*p* < 0.20) to identify independent variables to be considered in the main effects model [24]. Next, the multivariable model was built by iteratively considering independent variables in the model and comparing full and reduced models using partial F-tests [24]. Additionally, pair-wise interactions for all the independent variables in the main effects model were created and tested for significance. Previous research has established a clear relationship between mental health outcomes (including stress) and gender and age [26,27,28]. Based on this previous work, we considered both age and gender confounding variables, and as such they were both included in the final model regardless of statistical significance. The fit of the final multivariable model was examined using several graphical techniques, including scatterplots to assess Cook’s Distance, DFITS (difference in fits), leverage, and outliers [24]. If any of these tests suggested an observation was significantly influencing the model, it was examined for accuracy (e.g., data entry error). If data were accurate and plausible, the observation remained in the model. Model assumptions were examined using the Cook-Weisberg test for heteroskedasticity and the Shapiro-Wilk W test for normal data [24]. If data were not normally distributed, appropriate transformations would be assessed for best model fit. Statistical significance was assessed using *p* < 0.05 unless otherwise specified. Based on the results of the model, qualitative data were analyzed to provide context and depth to any identified risk and/or protective factors associated with PSS score.

### 2.3. Qualitative Methods

#### 2.3.1. Interview Recruitment

The qualitative data pertaining to stress in this study arose from a larger farmer mental health project involving semi-structured in-depth qualitative interviews [29] with farmers and people who work with farmers (e.g., agricultural bankers, crop advisors, agricultural insurance providers). Participants who were not themselves farmers but worked with farmers in their daily roles were identified by the community (farmers) as key informants to interview around the stresses they see in the farmers that they worked with. Based on this community recommendation, their perspectives were included. Potential participants were sent a recruitment poster via email through our agricultural stakeholder working group (which included farmers across commodities and other agricultural industry members). The first 75 eligible participants to contact the research team were interviewed. Interviews were conducted from July 2017–May 2018. Farmers located within 200 km of the University of Guelph (located in southwestern Ontario, Canada) were interviewed in person, at a location of their choosing. Those located >200 km from the University of Guelph were interviewed via telephone. Topics of discussion related to the present study included farming stresses and their mental health impacts; personal well-being; and resilience. Interviews lasted 45–75 min each and were audio-recorded and professionally transcribed. Transcriptions were checked against the audio recordings for accuracy by the research team. A short demographic survey was completed by the participant before the interview.

#### 2.3.2. Interview Participants

Seventy-five participants completed an in-depth interview. The demographic survey was completed by 74 of the 75 participants. Participants ranged from 25–78 years of age; 37 were men (50%) and 37 women (50%). Self-reported employment included farmers (51/74; 69%), agricultural industry staff (14/74; 19%), veterinarians (6/74; 8.1%), and one participant (1.4%) each from agricultural government, academia, and journalism. Most interviews were completed in person (65/75; 86.7%), and ten interviews (13.3%) were conducted over the phone.

#### 2.3.3. Interview Data Analysis

To provide depth and context to the quantitative results, we analyzed the qualitative interview data to explore farmers’ stress and the factors associated with stress. Thematic analysis, as described by Braun and Clarke (2006), was conducted collaboratively by three authors (B.H., A.S., A.J.B.). Quirkos^©^ (Quirkos, Edinborough, UK) data analysis software was used to facilitate the analysis [30]. Analysis involved using a combination of data-driven (inductive) and deductive approaches [31,32] and included the following steps: (a) familiarization with transcripts; (b) open-coding (i.e., freely coding without use of a codebook) of full transcripts in sections [30,32,33]; (c) production of a codebook outlining codes, their meanings, and exemplar quotations [30,33]; (d) coding all transcripts with the codebook [30,33]; (e) grouping codes to produce themes [30,31,32]; (f) reviewing and refining themes (including checking for supporting and disconfirming evidence) [34]; and (g) naming and defining of themes [30]. Verbatim explanatory quotes were selected to provide depth and context around how factors related to farming may impact a farmer’s level of perceived stress. Data reliability and authenticity techniques included using a collaborative approach to data analysis, a detailed audit trail, and a rich, detailed description of the results [35]. The collaborative approach included having multiple coders on each transcript, along with the member-checking of codes and quotes by members of the farmer community. The audit trail included detailed notes on the analysis process by the coders, including individual notes, analysis meeting notes, and member-checking meeting notes.

## 3. Results

### 3.1. Multivariable Linear Regression Model Results

**Figure 1:**
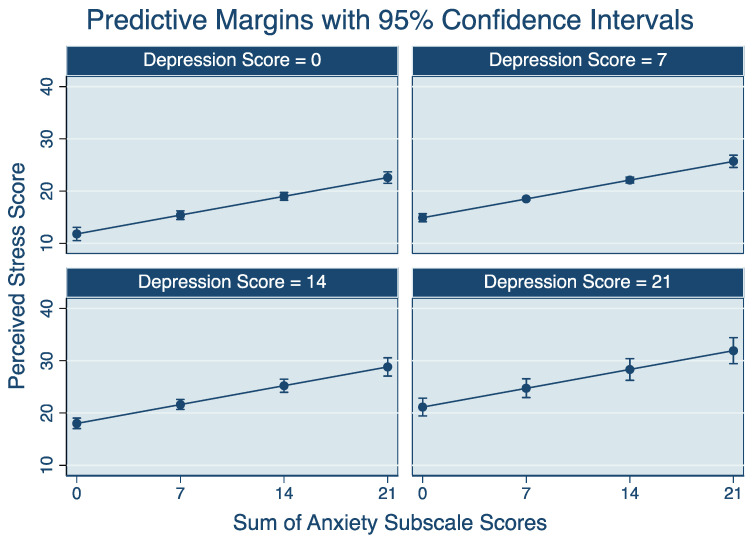
Predicted effects of the Hospital Anxiety and Depression Scale (HADS) anxiety score on Perceived Stress Scale Scheme 1. *Note:* Depression scores, according to the Hospital Anxiety and Depression Scale cutoffs, are as follows: 0 = no caseness, 7 = mild caseness, 14 = moderate caseness, and 21 = severe caseness.

Results from the final multivariable model of perceived stress score are displayed in Table 1. The adjusted R-squared for this model was 0.644. Participants who were experiencing financial stress scored higher on the PSS compared to participants who reported no financial stress. As financial stress scores increased from “a little” (0.779 points higher) to “some” (1.004 points higher) to “a lot” (2.300 points higher), the magnitude of the association with perceived stress score increased. Participants who identified as women scored 0.554 points higher than men on the PSS. Participants who farmed pigs scored significantly higher than all other commodities on the PSS. Participants who reported being dissatisfied with the support they received from their family and from their industry were more likely to report higher PSS compared to participants who reported being satisfied with these supports. For each 1-point increase in resilience score, the PSS score significantly decreased by 0.044 points. There was a significant interaction between anxiety and depression, such that the association of anxiety score with PSS depended on the depression score (and vice versa). Regardless of depression score, PSS increased with increasing anxiety scores. This increase in PSS was more pronounced with each increase in anxiety score. Similarly, regardless of anxiety score, PSS increased with increasing depression scores, and this increase in PSS was more pronounced with higher anxiety scores. The relationships of anxiety and depression to PSS score are shown graphically in Figure 1.

Model diagnostics confirmed that our model fit the data. Using the standardized residuals from the model, the results of the Cook-Weisberg test for heteroskedasticity revealed that the model was homoscedastic (*p* = 0.773), and the Shapiro-Wilk W test for normal data indicated that the data were normal (*p* = 0.20).

### 3.2. Qualitative Results

Seventy-four participants completed a pre-interview demographic form. Participants included 37 women (50%) and 37 men (50%). Self-reported employment of the participants included farmers (51/74; 69%), agricultural industry staff (14/74; 19%), veterinarians (6/74; 8.1%), agricultural government representatives (1/74; 1.4%), agricultural academics (1/74; 1.4%), and agricultural journalists (1/74; 1.4%). The ages of the participants ranged from 25–78 years. Sixty-five interviews were done in person, and 10 were done via telephone. In this section, we present the qualitative results that provide additional context and meaning to the variables found to be associated with perceived stress score in the multivariable model.

#### 3.2.1. Negative Impact of Financial Stress on Perceived Stress

Consistent with the model presented in Table 1, financial stress was described by participants as “a major uncertainty” and a concern related to farming that impacted their overall stress. As one participant explained,

“*…with us, our stress was almost all based on the financial struggles we were in. So we knew if we could get things turned around, we would all feel better… but there was nothing we could do at that time to make that happen.*”

Participants described financial stress in a number of different ways, with many conveying a high degree of severity. For example, one person explained that they “have one shot each year to make money”, and another said that “nowadays, farming is tantamount to taking a vow of poverty”. For many, there was a bleak outlook for farm finances, and this added to their overall stress. As one participant shared,

“*Where is it going to stop? When is the price of the land going to stop [increasing] if everything else doesn’t go up, you know? Cost of living goes up, but we don’t get more for our crops or now for our eggs, or if anything, well, they want those prices to go down. Well, how can the prices of the food that we’re producing go down, and we’re not getting paid any more for it, but everything else for us has gone up? How are we supposed to make a living with that, you know? And it’s just really hard to deal with all that. It’s just really, really hard, disheartening... It’s a lot of pressure, a lot of pressure.*”

Financial stress ranged from concerns regarding day-to-day expenses around “losing just one animal”, along with “costs of medication, and time, and vet bills”, to managing the stress of large amounts of debt required to run a farm and having “everything riding” on the farm’s financial success. For example, one participant described “…the debt load that they have, you can’t afford to make even small mistakes ‘cause there’s such a fine line between profitability and losing money”.

Livestock farmers explained how financial stress stemmed from the fact that raising livestock required financial flexibility because “animals are not widgets”, but this reality was incongruent with the inflexible “uniformity of production” required to “keep above those margins of profit”. Furthermore, many participants described how underlying financial stress increased the stress associated with making important decisions for animal health. This was a complicated construct, which one participant described as follows:

“*[There’s] what market demands force you into and what you know is right, and trying to do the best job you can for the animals you’re working with, but also being realistic about what you have to do to just make it work [financially].*”

Overall, many participants described that financial stress was “a constant” in most farmers’ lives and that it was a major contributor to their overall perceived stress; sometimes even when they were not consciously aware of it, it slowly diminished their mental health. As one farmer described,

“*The thing with the financial stress and the producer’s health is that they don’t even understand how it’s slowly ratcheting up their stress level and how that almost clouds, it almost carried forward with them.*”

#### 3.2.2. Relationship between Gender and Perceived Stress

Consistent with the multivariable model findings, women participants in the interviews described how being a woman farmer added to their overall stress. Women participants described the stress associated with “still feel[ing] like I’m a woman in a man’s world” and needing to work harder to establish themselves as the “actual farmer” versus the traditional label of “just the farm wife”. For example, some women described experiencing stress in having their identity as a “farmer” challenged, with several participants describing instances when someone (e.g., agronomist, salesperson) would come to the farm and immediately dismiss them by asking for “the farmer”. Additionally, participating women described stress (stemming from other feelings like frustration or anger) when trying to join industry groups or meetings and feeling dismissed or undervalued by agricultural community members.

Women participants also described stress stemming from bearing the load of multiple roles, like the “the stress of being a mom and a farmer”. These participants described having all the usual stress and stressors associated with being a farmer, but having the additional pressure and responsibility of childcare, child rearing, and household management loaded on top of their farming duties. For instance, one participant explained, “I deal with all the house stuff as well as the farm stuff. That’s also part of my job”. The necessity of needing to “balance everything” was an added stress for many participants who were mothers. For example,

“*For me as a mom, it’s really balancing everything. The children, all of their activities, [farm] chores. Especially—we rent three other farms. So the animals are at different locations, so dragging the kids with me when it’s −30 °C or whatever, right? And especially when they [the children] were smaller, it was quite an ambitious task.*”

One participant described this increased workload and the associated stress as follows:

“*I said [to farmer husband], do you know their [children’s] shoe sizes? Do you know what size clothes they’re wearing? Do you know those numbers? My head can only hold so much, right? But now, yeah, I still have to know all those numbers and now I know the barn numbers, the field numbers.*”

Participating women often discussed the burden of the additional roles they bore as part of being “mom” or “the wife”, and while those roles were often welcomed, they were not without struggle. For example, one woman participant described taking on a central role of worry in relation to the people on the farm:

“*I think, as a mom, a wife, you worry about your other family members. Are they too tired, are they too stressed, are they getting enough rest that they’re awake and aware of everything that’s happening around them, because accidents can happen so quickly on a farm.*”

Other women explained needing to be the “rock” for the family and that there was stress from needing to constantly fill that role and be “the cheerleader” for everyone in the family and on the farm. For example,

“*…my children see that mom’s down. Wow, ‘it’s bad when mum is struggling’. And they can see me struggling, and I have an open relationship [with them], we all do with each other, and so they see it and then it sort of rocked their whole world because, yeah, our families dynamics, that hasn’t been a part of them before.*”

Overall, the qualitative results support the quantitative results related to gender. Women participants described additional stressors and stresses that arose from traditional gender roles and their need to find a rightful place in agriculture.

#### 3.2.3. Relationship between Perceived Industry Peer Support and Perceived Stress

Results from the multivariable model showed that there was a positive association between a lack of perceived support from the industry and perceived stress. Within the qualitative analysis, while many participants spoke favorably about being part of the agricultural industry and the support they felt (e.g., that farmers are “very neighborly and have a very good social network”), they also described considerable stress associated with living in a small community where “everyone knows everyone’s business” and where farmers perceive a societal expectation that they are “not supposed to show any weakness”. One farmer explained,

“*And I think the other problem with stress is farmers don’t want to show weakness because we have to interact with the community and so—And I think I’ve seen it myself that if word gets out that you’re having financial stress on a farm, then your suppliers don’t want to deal with you as much, your bankers start to pull back, and so you get this kind of isolation going on.*”

Given these community dynamics and perceived (agri)cultural expectations, some participants also described how much of their stress came from “the overwhelming stress” of “continuously comparing” themselves to other farmers and from farmer-to-farmer competitiveness. It was common for participants to describe a sense of constantly “being watched” by members of the agricultural community, which intensified their stress and decision-making for fear of “being judged”. One participant explained that “what farmers are doing is they’re fighting [other] farmers, and so they’re always competing against each other in a race to the bottom”. Another participant explained,

“*Farmers are in this weird situation that, yeah, they’ve got their next door neighbor they’ve known their whole life, and that’s a person they can trust, but on the other hand they don’t want to show weakness because that is actually another competitor in the marketplace, and so if they show weakness there, well then, they’ll use that to their advantage to maybe take over your farm.*”

#### 3.2.4. Relationship between Perceived Support from Family and Perceived Stress

Dissatisfaction with perceived support from family was positively associated with perceived stress score in the multivariable model and was a common theme in the qualitative analysis. A lack of support from family was often discussed in terms of succession planning. Succession planning was described as “one of the biggest stresses out there” and intensified participants’ overall level of perceived stress. Stories of family conflict and/or complete breakdown around succession planning and the (lack of) transition of farm ownership and control to younger generations were common. As one farmer explained,

“*There’s so many farmers that hold on to everything pretty tight until right ‘til the end, and their sons mostly never really get a chance to own anything until it’s almost too late, and then maybe dad dies and plans aren’t made, and next thing you know, they lose that farm…*”

Separate from succession planning, participants also discussed the complexity of working directly with family, who they are with “day in and day out”, “twenty-four/seven”. While many participants explained that working with family “can be a very positive thing”, there can also “be a lot of animosity with family”. Many participants described how working with family can increase overall stress because “there is no escape valve” for stress when “you live with these same people [that you work with] day in and day out”. For example, one participant explained,

“*Family dynamics gets into the way as well, because often time you’re working with your brother, and no matter if you’re a farmer or whoever—you don’t always get along with your brothers or your sisters. Or if you’re working close with your dad that can be stressful too because you may be of similar personality, and clash. And yeah, there’s the whole personal interaction of people and it’s not like you’re just going to work and you get to say goodbye at 5:00, right? You live with these people and they’re there around the clock, so it can be stressful and it can be very stressful.*”

In several instances, the extent of the familial farming stress led to the splitting of farms. For example, one respondent commented, “we hated farming with family, but now we don’t do that, so that was a huge stress that we just got rid of ‘cause that was awful”.

When participants described a lack of support from family, stemming from issues with succession planning or interpersonal dynamics, it was often described as having a compounding effect on their stress overall.

#### 3.2.5. Positive Impact of Resilience on Perceived Stress

While we did not ask participants to define resilience, participants described their resilience in many ways. Some referred to a forced resilience or stoicism from the societal expectation that farmers are innately “supposed to be strong, and resilient, and suck it up, and ride through anything, and be salt of the earth”. Other participants described the work that goes into remaining resilient by leaning on family members and “venting” about their stress in order to “alleviate it and remain resilient”. Some participants said that they had “a very strong bond with the land” or their animals and that taking time to “connect with the land and their farms” helped them to build resilience against stress. One participant described how this “bond with the land” actually allowed them to “become aware of [their] own stress”. Furthermore, many participants discussed coping strategies around “creating boundaries” to distance themselves from the farm while they are “off duty”, even while remaining on the farm, as a way to build resilience and manage stress.

Finally, many participants felt a strong sense of “meaning and purpose” on their farms, bringing them “fulfillment”, and described that, in times of high stress, reflecting on this purpose helped them draw upon their resilience. One participant commented, “we take a lot of pride in the fact that we feed people. That it’s work that you feel is meaningful”. In support of the multivariable model results, participants described that drawing on their resilience skills (through myriad ways) served to decrease their overall stress.

## 4. Discussion

This study used a sequential explanatory mixed-methods approach to explore the factors associated with perceived stress among Canadian farmers. Results of the multivariable model indicated that financial stress at any level (“a little”, “some”, or “a lot”), pig farming, gender, and dissatisfaction with perceived family and industry supports was positively associated with participants’ perceived stress scores. There was a negative association between resilience scores and perceived stress scores, and participants’ depression and anxiety scores had a significant modifying effect on PSS scores.

It is worth noting that while the model findings were statistically significant, with the exception of financial stress, the effect sizes were generally small in magnitude (with model coefficients ranging from 0.02 to 1.2, or 0.05% to 3%, of the 40-point PSS). The subsequent qualitative analysis explored perceived stress more in depth, which supported the model findings and provided insights into the complexity of the factors that were not possible to capture within the survey.

The significant association found between financial stress and perceived stress is consistent with previous studies. In India, agricultural drought resulted in an economic collapse, which was strongly associated with greater financial strain and psychological stress [36]. A 2003 study conducted in the United States reported that, among farmers, financial stress and economic hardship were significantly associated with increased stress [37]. Additionally, a qualitative analysis conducted among Australian farmers identified financial pressure as a dominant source of increased stress [38]. In the present study, there was a dose-response relationship between financial stress and PSS; with increased levels of financial stress associated with increased PSS scores. The qualitative analysis within our study explored financial stress in depth; financial stress was found to be inherently tied up with numerous other factors, each of which also contributed to overall stress. For example, financial stress itself was related to the uncertainties of farming overall as well as with the perceived support that farmers reported as receiving from their industry. To illustrate this point, the competitiveness within the industry/agricultural community described in the qualitative analysis often revolved around financial competition, which in turn had a compounding effect, increasing perceived stress among participants. By way of another example, issues related to succession planning were described by interview participants as being related to feelings of lack of family support, but also had implications for financial stress, compounding participants’ overall perceived stress. These complexities of “financial stress” may be difficult to accurately capture within a quantitative survey alone, highlighting the usefulness of the qualitative exploration of factors impacting financial stress among farmers.

Within the multivariable model, pig farming was the only commodity significantly associated with higher scores on the PSS, when controlling for all other variables in the model. Previous research conducted in Canada has reported that livestock farmers scored significantly higher on chronic stress as compared to crop farmers [39]; however, commodity specific analyses were not conducted. The data for this study came from a cross-sectional survey that took place at a time when Canada’s pork industry had just experienced a Porcine Epidemic Diarrhea (PED) virus outbreak. The Ontario Ministry of Agriculture and Rural Affairs reported that the last case of PED was reported on 14 July 14 2015 [40]. It may be that the stresses associated with PED (e.g., financial loss [41] and animal loss [42]) impacted the overall perceived stress score for pig farms. We did not observe differences in perceived stress by commodity in the qualitative analysis, though the study was not designed or intended for that purpose.

Women scored higher on the PSS compared to men. This finding is consistent with a previous Canadian study that examined the differences in the level of stress between farmers and non-farmers [39]. In that study, within the farming group, women scored significantly higher on stress compared to men; within the non-farming group, there was no difference between genders. Other studies have not reported significant differences in levels of stress by gender among farmers, though much of the focus of the literature in high-income countries is on men [13]. One study in the United States reported that while men and women farmers experienced stress differently, particularly around childcare, where women reported inadequate childcare as more stressful than men, these findings were not statistically significant [43]. Another study, conducted in 2012 in Australia, also reported no statistically significant differences in levels of psychological stress among farming men and women [44]. Nevertheless, in our qualitative analysis, women provided descriptions of unique factors associated with increased stressed due to being a woman farmer. Some of these qualitative descriptions were directly reflective of other aspects of the model results, including a perceived lack of support from their industry and families, in terms of feeling “left out” at industry meetings and under-supported by family in terms of division of household duties. Women participants also described feelings of overwhelm from intensely gendered workloads, making them responsible for many household as well as farm duties, which contributed to their overall stress. Additionally, there was increased stress that women farmers associated with constantly “fighting” for their place “as a farmer” within the broader agricultural community. This struggle included advocating for roles within their industry organizations, for example, and contributed to increased stress described by women participants. Previous research has established that women who enter careers that have been traditionally male dominated leave those jobs quickly and more frequently than women who take on traditionally female-dominated roles [45]. One grounded-theory analysis in South Africa reported that women experienced inadequate accommodations from workplaces, making traditionally male-dominated careers inaccessible to many women [46]. Similar to the results of our study, accommodations around work– life balance, physical demands, and gendered discrimination were described by women in that study as important factors that impacted their stress and their decisions to leave their jobs [46]. According to the latest available data (2016), approximately 28% of farms in Canada are led by women [47]. While the number of farmers in Canada is decreasing overall [48], finding ways to incorporate accommodations that will encourage women farmers to enter farming, feel they rightly belong, and thus remain in this occupation are important. Stress management and well-being interventions targeted towards women farmers could also be a useful avenue to explore.

Previous research has established positive association between an individual’s level of stress and the onset of depression and anxiety [49]; therefore, the results observed here with respect to anxiety and depression were expected and consistent with previous research [49]. More specifically, the significant interaction in our model was additive, meaning that higher anxiety and depression scores exacerbated the magnitude of the PSS score among participants. Similarly, a study with farmers in the United States reported that higher levels of stress were associated with increased depression [36].

Beyond the factors identified in the model results, the qualitative analysis also allowed for an in-depth exploration of additional factors that impact perceived stress among Canadian farmers, though reporting them here is beyond the scope of this article. The qualitative analysis also provided the opportunity to explore factors that may contribute to the unidentified variability within the quantitative data by disentangling complex constructs, like financial stress. Using the sequential explanatory design allowed for a more comprehensive understanding of how the cultural context of farming permeates constructs such as financial strain and gender. Further use of mixed methods in the investigation of complex constructs like farmer stress and mental health is recommended.

### Limitations

Due to the cross-sectional design of the survey, we cannot infer any time component for the independent variables. Therefore, it is possible that the independent variables were a result of the outcome, rather than influencing the outcome. Additionally, due to the convenience sampling method used to collect the survey data, we have limited ability to extrapolate these results to a wider population. However, the qualitative analysis that supplements the model results served to provide insights into areas for future investigations of perceived stress among farmers.

## 5. Conclusions

This study used quantitative and qualitative methods in a mixed-methods approach to provide a comprehensive exploration of factors that impact perceived stress among Canadian farmers. Factors associated with increased perceived stress scores included financial stress, pig farming, woman gender, and dissatisfaction with perceived supports from family and industry, as well a depression and anxiety (in an interaction term). There was a negative association between resilience and increased perceived stress. The results from the qualitative analysis gave support to, provided depth to, and expanded upon, the model findings. The insights into farmer stress provided by this study support the need for, and can help inform, stress management approaches and interventions for farmers (particularly women). The findings can also inform future areas of investigation of farmer stress and mental health by being based in participating farmers’ lived experience.

## Figures and Tables

**Table 1:** Results of a multivariable linear regression model of Perceived Stress Scale score and associations with age, gender, financial stress, satisfaction with family and industry support, resilience, depression, and anxiety scores among 914 Canadian farmers (2015–2016).

	Coefficient ^1^	95% Confidence Interval	*p*-Value
Age ^2^ (years)	−0.012	−0.027, 0.003	0.117
Gender (ref: men)	0.554	0.118, 0.990	0.013
Financial Stress (ref: none)			
*A little*	0.779	0.127, 1.431	0.019
*Some*	1.004	0.353, 1.657	0.003
*A lot*	2.300	1.590, 3.004	<0.001
Dissatisfied with family support (ref: satisfied)	1.183	0.389, 1.977	0.004
Dissatisfied with industry support (ref: satisfied)	1.148	0.156, 2.140	0.023
Farms Pigs	1.065	0.446, 1.685	0.001
Resilience Score	−0.044	−0.061, −0.027	<0.001
DepressionXAnxiety Interaction ^3^	−0.017	−0.027, −0.007	0.001
Depression Score	0.445	0.322, 0.567	<0.001
Anxiety Score	0.514	0.424, 0.604	<0.001

*Note:* ^1^ A coefficient = 1 represents a 1-point change in the PSS scale (out of a possible 40) ^2^ Age and gender were considered potential confounding variables and were forced into the model regardless of significance. ^3^ The interaction between depression score and anxiety score and how it is associated with perceived stress is reported visually in Figure 1. Variables excluded from the final model include farm ownership, how rural a farm was (kilometer distance from urban center), self-rated health and mental health, past mental illness, and perceived satisfaction with support from their spouse/romantic partner and friends.

## Data Availability

The data presented in this study are available on request from the corresponding author. The data are not publicly available in order to protect the participants identity.
